# HunFlair: an easy-to-use tool for state-of-the-art biomedical named entity recognition

**DOI:** 10.1093/bioinformatics/btab042

**Published:** 2021-01-28

**Authors:** Leon Weber, Mario Sänger, Jannes Münchmeyer, Maryam Habibi, Ulf Leser, Alan Akbik

**Affiliations:** Computer Science Department, Humboldt-Universität zu Berlin, Berlin 10099, Germany; Group Mathematical Modelling of Cellular Processes, Max Delbrück Center for Molecular Medicine in the Helmholtz Association, Berlin 13125, Germany; Computer Science Department, Humboldt-Universität zu Berlin, Berlin 10099, Germany; Computer Science Department, Humboldt-Universität zu Berlin, Berlin 10099, Germany; Section Seismology, GFZ German Research Centre for Geosciences, Potsdam 14473, Germany; Computer Science Department, Humboldt-Universität zu Berlin, Berlin 10099, Germany; Computer Science Department, Humboldt-Universität zu Berlin, Berlin 10099, Germany; Computer Science Department, Humboldt-Universität zu Berlin, Berlin 10099, Germany

## Abstract

**Summary:**

Named entity recognition (NER) is an important step in biomedical information extraction pipelines. Tools for NER should be easy to use, cover multiple entity types, be highly accurate and be robust toward variations in text genre and style. We present *HunFlair*, a NER tagger fulfilling these requirements. HunFlair is integrated into the widely used NLP framework *Flair*, recognizes five biomedical entity types, reaches or overcomes state-of-the-art performance on a wide set of evaluation corpora, and is trained in a cross-corpus setting to avoid corpus-specific bias. Technically, it uses a character-level language model pretrained on roughly 24 million biomedical abstracts and three million full texts. It outperforms other off-the-shelf biomedical NER tools with an average gain of 7.26 pp over the next best tool in a cross-corpus setting and achieves on-par results with state-of-the-art research prototypes in in-corpus experiments. *HunFlair* can be installed with a single command and is applied with only four lines of code. Furthermore, it is accompanied by harmonized versions of 23 biomedical NER corpora.

**Availability and implementation:**

*HunFlair* ist freely available through the *Flair* NLP framework (https://github.com/flairNLP/flair) under an MIT license and is compatible with all major operating systems.

**Supplementary information:**

Supplementary data are available at *Bioinformatics* online.

## 1 Introduction

Recognizing biomedical entities (NER) such as genes, chemicals or diseases in unstructured scientific text is a crucial step of all biomedical information extraction pipelines. The respective tools are typically trained and evaluated on rather small gold standard datasets. However, in any real application they are applied ‘in the wild’, i.e. to a large collection of texts often varying in focus, entity distribution, genre (e.g. patents versus scientific articles) and text type (e.g. abstract versus full text). This mismatch can lead to severely misleading evaluation results. To address this, we recently released the *HUNER* tagger (Weber et al., 2020) that was trained jointly on a large collection of biomedical NER datasets, leading to a much better performance on unseen corpora compared to models trained on a single corpus. However, *HUNER* relies on a Docker installation and uses a client-server architecture. These design decisions do not hinder its own installation but make its integration into any of the major NLP frameworks, which is required for the construction of comprehensive information extraction pipelines, cumbersome. Moreover, *HUNER* does not build upon a pretrained language model (LM), although such models were the basis for many recent breakthroughs in NLP research (Akbik *et al.*, 2019).

Here, we present *HunFlair*, a redesigned and retrained version of *HUNER* integrated into the widely used *Flair* NLP framework. *HunFlair* builds upon a pretrained character-level language model. It recognizes five important biomedical entity types with high accuracy, namely *Cell Lines*, *Chemicals*, *Diseases*, *Genes* and *Species*. Through its shipping as a Flair component, it can be easily combined with other IE tools (e.g. text parsing, document classification, hedge detection) or other language models and benefits from the experiences and future developments of the large user and developer base of *Flair*. Through its simple but extensible interface, it is easily accessible also for non-experts. Technically, *HunFlair* combines the insights from Weber *et al.* (2020) and Akbik *et al.* (2019) by merging character-level LM pretraining and joint training on multiple gold standard corpora, which leads to strong gains over other state-of-the-art off-the-shelf NER tools. For *HunFlair*, we specially trained a character-level in-domain LM on a large corpus of biomedical abstracts and full-texts and make it publicly available to facilitate further research.

In addition, we integrate 23 biomedical NER corpora into *HunFlair* using a consistent format, which enables researchers and practitioners to rapidly train their own models and experiment with new approaches within *Flair*. Note that these are the same corpora that were already made available through *HUNER*. However, the integration into *Flair* has the additional benefits of more convenient automated downloading and flexible preprocessing. While *HUNER*’s corpora came preprocessed with a particular method, users of *HunFlair* may process the corpora along with their own choices, for instance by using different sentence resp. word segmentation methods.

## 2 Hunflair

*HunFlair* was created by implementing the approach behind *HUNER* into the *Flair* NLP framework, along with its improvement by integrating a pretrained language model. *Flair* is an NLP framework designed to allow intuitive training and distribution of sequence labeling, text classification and language models. *Flair* achieves state-of-the-art performance in several NLP research challenges (Akbik et al., 2018), allows researchers to ‘mix and match’ various types of character, word and document embeddings and features a base of more than 120 contributors. In addition, more than 500 open-source projects and python libraries rely on Flair (see https://github.com/flairNLP/flair).

**Fig. 1.**
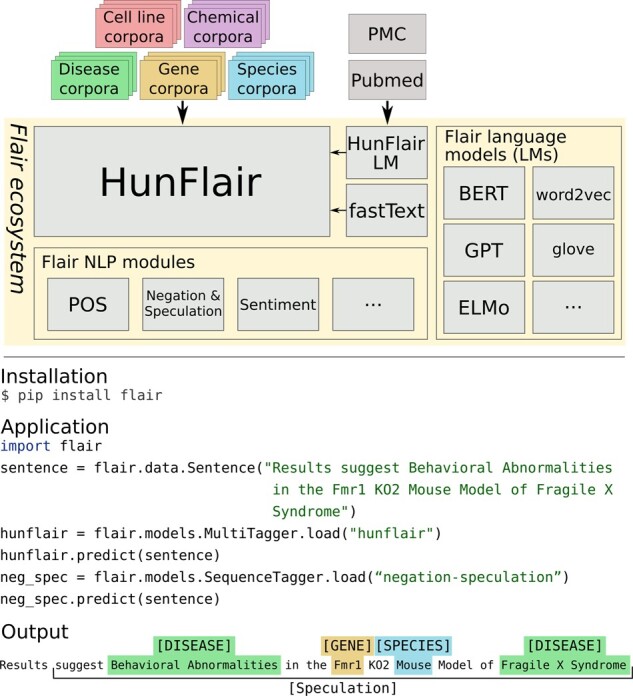
Overview of the *HunFlair* model and it’s integration into the Flair ecosystem. The model is based on a biomedical Flair character-level language model and word embeddings from fastText. In total, the model was trained on 23 biomedical NER datasets spanning five distinct entity types. Furthermore, the simple installation and application of *HunFlair* as well as it’s integration with other Flair components is shown exemplarily

Figure 1 shows the architecture of *HunFlair* and illustrates how little coding is required to use it. At the core, it relies on a Flair character-level language model trained on roughly 24 million abstracts of biomedical articles from PubMed and 3 million full texts originating from PMC as well as fastText word embeddings (Bojanowski et al., 2017). The inclusion of such pretrained character-level language models in NER models lead to strong improvements in other domains (Akbik *et al.*, 2018). Prediction of named entities is performed by a BiLSTM-CRF model (Huang et al., 2015). Following the HUNER approach, it consists of distinct models for each entity type which are trained on the union of all training sets of all integrated gold standard NER corpora with this type to achieve a more robust performance across other texts, text genres and biomedical sub-domains. See Supplementary Material S1 for details of the training process.

## 3 Results

We compare the tagging accuracy of *HunFlair* to two types of competitors: Other ‘off-the-shelf’ biomedical NER tools, and other recent research prototypes. Therefore, we classify a tool as off-the-shelf when it (i) comes with pretrained prediction models (ease of use), and (ii) can be locally installed (to allow the application to potentially large and potentially propriatary text collections). In contrast, we classify a tool as research prototype when its application requires a retraining of models or when it is only usable as a web service.

Our primary comparisons to off-the-shelve tools are based on cross-corpus experiments, because these give insight into the generalization properties of a model across different text types (e.g. full text versus abstract) and scientific subdomains (e.g. human oncology, psychological diseases, biology of plants, etc.). Clearly, this comes at the price of introducing a bias against methods which were designed for specific annotation guidelines that differ from those of an evaluation corpus. Therefore, our comparisons to research prototypes are based on in-corpus experiments which evaluate the architecture of *HunFlair* also in this setting.

### 3.1 Comparison to off-the-shelf tools

We compare the performance of *HunFlair* in a cross-corpus setting to five other state-of-the-art biomedical NER tools using three gold standard corpora: CRAFT (Bada et al., 2012), BioNLP13 Cancer Genetics (Pyysalo et al., 2013) and PDR (Kim et al., 2019). None of these was used in the training of neither *HunFlair* nor any competitor tools and we checked that there are no significant textual overlaps between these corpora and any of *HunFlair’*s trainings corpora. We compare (restricted to the supported entity types) against *SciSpacy* (Neumann et al., 2019), *HUNER* (Weber *et al.*, 2020), *tmChem* (Leaman et al., 2015), *GNormPlus* (Wei et al., 2015) and *DNorm* (Leaman et al., 2013). As *SciSpacy* comes with several models for each entity type, we report the best performance among all of those models that were not trained on the evaluation corpus. Results can be found in Table 1.

**Table 1.** F1-scores of several off-the-shelf biomedical NER tools on three unseen corpora

	CRAFT			BioNLP CG				PDR
	Ch	G	S	Ch	D	G	S	D
Misc	42.88	64.93	81.15	72.15	55.64	68.97	**80.53**	80.63
SciSpacy	35.73	47.76	54.21	58.43	56.48	66.18	57.11	75.90
HUNER	42.99	50.77	84.45	67.37	55.32	71.22	67.84	73.64
HunFlair	**59.69**	**72.19**	**85.05**	**81.82**	**65.07**	**87.71**	76.47	**83.44**

*Note*: We distinguish entity types Chemical (Ch), Disease (D), Gene (G) and Species (S). The best results are in bold. Misc displays the results of multiple taggers: *tmChem* for Chemical, *GNormPlus* for Gene and Species and *DNorm* for Disease. Note, this cross-corpus evaluation setup aims to assess the robustness of the tools regarding varying entity type definitions. However, it can introduce a bias against tools designed for a particular annotation guideline such as those listed under *Misc*.

*HunFlair* outperforms all competitors in all but one comparison, with an average gain of 7.26 pp in F1. Note that this evaluation uses mention-level F1 scores and compares against the gold spans annotated in the original corpora, while allowing for a one-character offset which accounts for differences in the handling of special characters. Results for a slightly different evaluation protocol, which considers as match any overlap between gold standard and predicted spans, along with a more in-depth discussion of evaluation setups and results, can be found in Supplementary Material S2. Although especially *SciSpacy* and *HUNER* profit from this more lenient evaluation (+8.04 pp/+5.55 pp), the overall ranking of methods is not changed.

### 3.2 Comparison to research prototypes

We compare *HunFlair’s* results in an in-corpus setting to those reported by four different research prototypes based on three different corpora: JNLPBA (Kim et al., 2004), BioCreative V CDR (Li et al., 2016) and NCBI Disease (Doğan *et al.*, 2014). These corpora were chosen because of the availability of published results on the test splits. Specifically, we compare to *BioBERT* (Lee et al., 2019), *SciBERT* (Beltagy *et al.*, 2019), *CollaboNet* (Yoon et al., 2019) and *SciSpacy* (Neumann *et al.*, 2019). To ensure a fair comparison, we proceed as follows when evaluating *HunFlair* in this setting. We first remove the three evaluation corpora from the pretraining set. We next pre-train *HunFlair* on all remaining corpora and then fine-tune it on the training and development portions of the respective corpus.

The results can be found in Table 2 and the detailed evaluation protocol is described in Supplementary Material S3. *HunFlair* sets the new state-of-the-art on *BioCreative V CDR* consisting of chemical and disease annotations with a macro-average F1 score of 90.57. For JNLPBA (gene) and NCBI Disease, it reaches on-par results with the competitor methods. We also investigate the effect of pretraining on multiple gold standard corpora, by comparing *HunFlair* to a non-pretrained version on all 23 NER corpora. On average, finetuning improves results on all entity types with the improvements in F1 ranging from 0.8 pp for chemicals to 4.75 pp for cell lines. The full results per corpus are provided in Supplementary Material S4.

**Table 2.** Comparison with the reported results of state-of-the-art research prototypes for BioNER

	JNLPBA (Gene)	BC5CDR	NCBI
SciBERT	77.28	90.01	88.57
BioBERT v1.1	77.49	89.76	**89.71**
CollaboNET	**78.58**	87.68	88.60
SciSpacy	–	83.92	81.56
HunFlair	77.60	89.65	88.65
HunFlair (vanilla)	77.78	**90.57**	87.47

*Note*: Scores are macro-averaged F1 and best results are printed in bold. Note, that HunFlair has been trained on the training and development portions of the respective corpus. ’HunFlair (vanilla)’ refers to the HunFlair model without pretraining on gold standard corpora.

## 4 Conclusion

We proposed *HunFlair*, a state-of-the-art biomedical NER tagger. Through its tight integration into the Flair NLP framework, it is easy to install, easy to use and easy to combine with other NLP modules. It comes comes along with 23 biomedical NER corpora in a single format while still enabling customized pre-processing. *HunFlair* is a redesign of *HUNER*, which it extends with pretrained domain-specific character-level language models. It outperforms a series of other off-the-shelf tools in a cross-corpus evaluation setting on different datasets, and achieves on-par results with current state-of-the-art research prototypes based on in-corpus experiments.

## Funding

L.W. and J.M. were funded by the Helmholtz Einstein International Berlin Research School in Data Science (HEIBRiDS). M.H. was funded by the German Research Council [LE-1428/7-1].

*Conflict of Interest*: none declared.

## Supplementary Material

btab042_Supplementary_Data

## References

[btab042-B1] Akbik A. et al (2018) Contextual string embeddings for sequence labeling. In Proceedings of the 27th International Conference on Computational Linguistics. Association for Computational Linguistics, Santa Fe, New Mexico, USA, pages 1638–1649.

[btab042-B2] Akbik A. et al (2019) FLAIR: An easy-to-use framework for state-of-the-art NLP. In *Proceedings of the Conference of the NAACL 2019 Conference (Demo)*. Association for Computational Linguistics, Minneapolis, MN, pp. 54–59.

[btab042-B3] Bada M. et al (2012) Concept annotation in the craft corpus. BMC Bioinformatics, 13, 161. 22776079 10.1186/1471-2105-13-161 PMC3476437

[btab042-B4] Beltagy I. et al (2019) SciBERT: a pretrained language model for scientific text. In Empirical Methods in Natural Language Processing 2019 (EMNLP). Association for Computational Linguistics, Hong Kong, China, pp. 3615–3620.

[btab042-B5] Bojanowski P. et al (2017) Enriching word vectors with subword information. Trans. ACL, 5, 135–146.

[btab042-B6] Doğan R.I. et al (2014) NCBI disease corpus: a resource for disease name recognition and concept normalization. J. Biomed. Inform., 47, 1–10. 24393765 10.1016/j.jbi.2013.12.006 PMC3951655

[btab042-B7] Huang Z. et al (2015) Bidirectional LSTM-CRF models for sequence tagging. ***arXiv preprint arXiv:1508.01991***.

[btab042-B8] Kim B. et al (2019) A corpus of plant–disease relations in the biomedical domain. PLoS One, 14, e0221582. 31461491 10.1371/journal.pone.0221582 PMC6713337

[btab042-B9] Kim J.-D. et al (2004) Introduction to the bio-entity recognition task at JNLPBA. In *Proceedings of the International Joint Workshop on Natural Language Processing in Biomedicine and Its Applications*. Citeseer, Geneva, Switzerland, pp. 73–78.

[btab042-B10] Leaman R. et al (2013) DNorm: disease name normalization with pairwise learning to rank. Bioinformatics, 29, 2909–2917. 23969135 10.1093/bioinformatics/btt474 PMC3810844

[btab042-B11] Leaman R. et al (2015) tmchem: a high performance approach for chemical named entity recognition and normalization. J. Cheminf., 7, S3. 10.1186/1758-2946-7-S1-S3 PMC4331693 25810774

[btab042-B12] Lee J. et al (2019) BioBERT: a pre-trained biomedical language representation model for biomedical text mining. Bioinformatics, 36, 1234–1240. 10.1093/bioinformatics/btz682 PMC7703786 31501885

[btab042-B13] Li J. et al (2016) BioCreative V CDR task corpus: a resource for chemical disease relation extraction. Database, 2016, baw068. 27161011 10.1093/database/baw068 PMC4860626

[btab042-B14] Neumann M. et al (2019) ScispaCy: fast and robust models for biomedical natural language processing. In: 18th BioNLP Workshop and Shared Task. Association for Computational Linguistics, Florence, Italy, pp. 58–66.

[btab042-B15] Pyysalo S. et al (2013) Overview of the cancer genetics (CG) task of BioNLP shared task 2013. In: BioNLP Shared Task 2013 Workshop. Association for Computational Linguistics, Sofia, Bulgaria, pp. 58–66.

[btab042-B16] Weber L. et al (2020) HUNER: improving biomedical NER with pretraining. Bioinformatics, 36, 295–302. 31243432 10.1093/bioinformatics/btz528

[btab042-B17] Wei C.-H. et al (2015) Gnormplus: an integrative approach for tagging genes, gene families, and protein domains. BioMed. Res. Int., 2015, 1–7. 10.1155/2015/918710 PMC4561873 26380306

[btab042-B18] Yoon W. et al (2019) Collabonet: collaboration of deep neural networks for biomedical named entity recognition. BMC Bioinformatics, 20, 249. 31138109 10.1186/s12859-019-2813-6 PMC6538547

