# Correction to: HunFlair: an easy-to-use tool for state-of-the-art biomedical named entity recognition

**DOI:** 10.1093/bioinformatics/btad705

**Published:** 2023-11-24

##  

This is a correction to: Leon Weber, Mario Sänger, Jannes Münchmeyer, Maryam Habibi, Ulf Leser, Alan Akbik, HunFlair: an easy-to-use tool for state-of-the-art biomedical named entity recognition, *Bioinformatics*, Volume 37, Issue 17, September 2021, Pages 2792–2794, https://doi.org/10.1093/bioinformatics/btab042

In the originally published online version of this manuscript,

In **section 3.1 Comparison to off-the-shelf tools**, second paragraph, first sentence should read: “HunFlair outperforms all competitors in all but one comparison, with an average gain of 7.04 pp in F1. N” instead of: “HunFlair outperforms all competitors in all but one comparison, with an average gain of 7.26 pp in F1. N”. The last sentence should read: “Although especially SciSpacy and HUNER profit from this more lenient evaluation (+13.53 pp/+10.33 pp), the overall ranking of methods is not changed” instead of: “Although especially SciSpacy and HUNER profit from this more lenient evaluation (+8.04 pp/+5.55 pp), the overall ranking of methods is not changed”.

Table 1’s figures are errored. Table 1 should read:

**Table 1.** F1-scores of several off-the-shelf biomedical NER tools on three unseen corpora

	CRAFT			BioNLP CG				PDR
	Ch	G	S	Ch	D	G	S	D
Misc	41.56	63.83	80.99	68.85	49.33	66.78	**75.12**	73.23
SciSpacy	33.78	45.15	47.58	55.26	50.91	59.39	47.43	68.40
HUNER	41.15	45.45	84.26	63.18	47.52	65.69	61.15	66.95
HunFlair	**58.40**	**67.07**	**84.94**	**79.93**	**58.61**	**83.63**	71.45	**76.82**

instead of:

**Table 1.** F1-scores of several off-the-shelf biomedical NER tools on three unseen corpora

	CRAFT			BioNLP CG				PDR
	Ch	G	S	Ch	D	G	S	D
Misc	42.88	64.93	81.15	72.15	55.64	68.97	**80.53**	80.63
SciSpacy	35.73	47.76	54.21	58.43	56.48	66.18	57.11	75.90
HUNER	42.99	50.77	84.45	67.37	55.32	71.22	67.84	73.64
HunFlair	**59.69**	**72.19**	**85.05**	**81.82**	**65.07**	**87.71**	76.47	**83.44**

In the **Supplementary data** file, Table SM3 was also errored. Table SM3 should read:

**Table SM 3.** Cross corpus evaluation of off-the-shelf BioNER tools for the entity types Chemical (Ch). Disease (D). Gene (G) and Species (S) counting any overlap between the predicted and annotated span as a true positive. All scores are F1-measures and the best results are in bold. Delta shows the improvement over the more strict evaluation reported in the main text (Table 1). Misc displays the results of multiple taggers: tmChem for Chemical. GNormPus for Gene and Species. and DNorm for Disease.

	CRAFT			BioNLP CG				PDR
	Ch	G	S	Ch	D	G	S	D
Misc	44.86	67.52	82.02	74.36	60.04	71.06	**84.18**	86.95
Δ	3.30	3.69	1.03	5.51	10.71	4.28	9.06	13.72
SciSpacy	39.78	54.71	72.02	60.65	61.69	78.77	65.04	83.49
Δ	6.00	9.56	24.44	5.39	10.78	19.38	17.61	15.09
HUNER	46.84	65.27	84.66	72.00	59.74	79.58	71.43	78.49
Δ	5.69	19.82	0.40	8.82	12.22	13.89	10.28	11.54
HunFlair	**61.99**	**80.50**	**85.46**	**83.52**	**69.29**	**92.73**	80.15	**88.64**
Δ	3.59	13.43	0.52	3.59	10.68	9.10	8.70	11.82

instead of:

**Table SM 3.** Cross corpus evaluation of off-the-shelf BioNER tools for the entity types Chemical (Ch). Disease (D). Gene (G) and Species (S) counting any overlap between the predicted and annotated span as a true positive. All scores are F1-measures and the best results are in bold. Delta shows the improvement over the more strict evaluation reported in the main text (Table 1). Misc displays the results of multiple taggers: tmChem for Chemical. GNormPus for Gene and Species. and DNorm for Disease.

	CRAFT			BioNLP CG				PDR
	Ch	G	S	Ch	D	G	S	D
Misc	44.86	67.52	82.02	74.36	60.04	71.06	**84.18**	86.95
Δ	1.98	2.59	0.87	2.21	4.40	2.09	3.65	6.32
SciSpacy	39.78	54.71	72.02	60.65	61.69	78.77	65.04	83.49
Δ	4.05	6.95	17.81	2.22	5.21	12.59	7.93	7.59
HUNER	46.84	65.27	84.66	72.00	59.74	79.58	71.43	78.49
Δ	3.85	14.50	0.21	4.63	4.42	8.36	3.59	4.85
HunFlair	**61.99**	**80.50**	**85.46**	**83.52**	**69.29**	**92.73**	80.15	**88.64**
Δ	2.16	6.99	0.42	1.70	4.22	5.02	3.74	5.20

These emendations are outlined only in this correction notice to preserve the version of record.

