# Supplementary material for: HunFlair: an easy-to-use tool for state-of-the-art biomedical named entity recognition
Source: Bioinformatics. 2021 Jan 28;37(17):2792–4. doi: 10.1093/bioinformatics/btab042 (PMC8428609; doi:10.1093/bioinformatics/btab042)
Supplement: btab042_Supplementary_Data [file btab042_supplementary_data.pdf]

Supplement: HunFlair: An Easy-to-Use Tool for State-of-the-Art Biomedical Named Entity Recognition

Leon Weber, Mario Sänger,
Jannes Münchmeyer, Maryam Habibi, Ulf Leser, Alan Akbik

2020

1 Training of HunFlair

The training of *HunFlair* is a two-step process. First, the required word embeddings are trained on a large unlabeled corpus, which are then used in the training of the NER tagger on multiple manually labeled NER corpora.

1.1 Embeddings

We use two types of word embeddings for *HunFlair*, (I) *Flair* embeddings based on a character-level language model (LM) and (II) *fastText* embeddings (Bojanowski *et al.*, 2017).

We trained the Flair LM on a corpus of roughly 3 million full texts from the PubmedCentral BioC text mining collection[1] and 25 million abstracts of PubMed articles[2], yielding a corpus of roughly 14 billion tokens, which we divide into 1500 splits. For the training of fastText, we used the same corpus, which we enriched with the text of 6,062,172 wikipedia articles[3], adding another 2.6 billion tokens.

For the Flair embeddings, we use a single-layer LSTM with a hidden size of 2048 for each direction. Both LSTMs are trained with a sequence length of 300, a batch size of 256 and a split-wise patience for the learning rate annealing of 100. For the fastText embeddings, we train a skip-gram model with 200 dimensions and sample 10 negative examples per step. The rest of the hyperparameters are left at their default value.

[1] `ftp://ftp.ncbi.nlm.nih.gov/pub/wilbur/BioC-PMC/`, Version of 2019/05/24

[2] ftp://ftp.ncbi.nlm.nih.gov/pubmed/baseline, Version of 2019/12/16A

[3] `https://dumps.wikimedia.org/enwiki/latest/enwiki-latest-pages-articles.xml.bz2`, Version of 2020/05/06

1.2 Gold standard NER pre-training

In order to have a broad data basis, we harmonize 23 manually-curated, biomedical NER corpora for the training of *HunFlair*. The corpora include patents, abstracts and full-texts from scientific articles and are annotated with a variety of entity types. Table SM 1 gives an overview about the included corpora and highlights important statistics. We are using almost the same data sets as in *HUNER* (Weber *et al.*, 2019). The only difference is that, for *HunFlair*, we excluded the BioSemantics corpus (Akhondi *et al.*, 2014) because the large number of very long sentences significantly slowed down training and we didn't observe any performance improvements in preliminary experiments using it. Note, that the reported number of used corpora differs from Weber *et al.* (2019), because here, we count a corpus only once even if it contains multiple entity types.

We use the sentence splitter and a modified version of the tokenizer of the *en_core_sci_sm* model of *scispacy*[4] (Neumann *et al.*, 2019). In preliminary experiments, we evaluated different tokenization strategies for *HunFlair* but did not observe any significant differences. We train distinct models for each entity type, i.e. cell lines, chemicals, disease, gene / proteins and species, to achieve high quality results. For each type we only use corpora that contain annotations for the respective entity type to learn a type-specific model. We re-use the splits introduced by *HUNER* to form a training and validation split for each data set. Our training sets are built by taking the union of the *HUNER* train and test splits of each data set. The validation sets are given by the union of all *HUNER* validation splits. The former is used to train the models and the latter to select the best performing model.

We apply a bidirectional LSTM-CRF neural network to model the recognition of named entities as sequence labeling task. We represent input words using the *HunFlair* language model and fastText embeddings learned on in-domain texts (see Section 1.1). Building on this, a single layer Bi-LSTM with a hidden size of 256 is used to process the input sequence. Prediction of the output sequence, i.e. one IOBES label per word, is done using a CRF in the final layer. All models are trained for 200 epochs with an batch size of 32, an initial learning rate of 0.1, dropout of 0.5 and a patience of 3.

[4] `https://s3-us-west-2.amazonaws.com/ai2-s2-scispacy/releases/v0.2.5/en_core_sci_sm-0.2.5.tar.gz`

Table SM 1: Overview of the 23 biomedical NER corpora used to train HunFlair. For each corpus we report the text genre (patent (P) / scientific articles (SA)), text type (abstract (A) / full-text (FT)) as well as number of sentence, token, entity annotation statistics.

Corpora	Genre	Type	Sentences	Tokens	Entity Type	Annotations	Unique Ann.
BioCreative II GM (Smith *et al.* (2008))	SA	A	20,744	545,966	Genes / Proteins	24,453	16,046
BioCreative V GPRO (Pérez-Pérez *et al.* (2017))	P	A	35,277	1,558,687	Genes / Proteins	13,125	5,662
BioCreative V CDR (Li *et al.* (2016))	SA	A	14,464	345,648	Chemicals	15,828	2,712
					Diseases	12,931	3,281
BioInfer (Pyysalo *et al.* (2007))	SA	A	1,138	37,135	Genes / Proteins	4,408	1,357
CellFinder (Neves *et al.* (2012))	SA	FT	2,211	70,286	Cell Lines	367	63
					Genes / Proteins	1,572	706
					Species	462	43
CHEMDNER patent (Krallinger *et al.* (2015b,a))	P	A	48,744	1,558,182	Chemicals	65,238	20,529
CHEBI (Shardlow *et al.* (2018))	P	FT	13,088	423,731	Chemicals	24,124	6,816
					Genes / Proteins	7,140	1,871
					Species	3,841	884
CHEMDNER (Krallinger *et al.* (2015a))	SA	A	87,550	2,431,366	Chemicals	83,058	20,470
CLL (Kaewphan *et al.* (2016))	SA	A, FT	201	7,689	Cell Lines	341	309
DECA (Wang *et al.* (2010))	SA	A	5,454	147,874	Genes / Proteins	6,261	2,187
FSU-PRGE (Hahn *et al.* (2010))	SA	A	36,216	985,598	Genes / Proteins	59,521	15,912
Gellus (Kaewphan *et al.* (2016))	SA	A, FT	11,809	312,699	Cell Lines	650	210
IEPA (Ding *et al.* (2002))	SA	A	486	16,590	Genes / Proteins	1,117	139
JNLPBA (Kim *et al.* (2004))	SA	A	18,535	532,777	Cell Lines	3,831	2,250
					Genes / Proteins	30,263	8,964
Linneaus (Gerner *et al.* (2010))	SA	FT	17,593	504,261	Species	2,724	339
LocText (Goldberg *et al.* (2015))	SA	A	945	24,178	Genes / Proteins	1,930	717
					Species	276	37
miRNA (Bagewadi *et al.* (2014))	SA	A	2,456	64,897	Diseases	2,032	586
					Genes / Proteins	944	345
					Species	676	45
NCBI Disease (Doğan *et al.* (2014))	SA	A	7,308	179,849	Diseases	6,861	2,137
OSIRIS (Furlong *et al.* (2008))	SA	A	1,072	31,020	Genes / Proteins	957	355
S800 (Pafilis *et al.* (2013))	SA	A	6,421	165,451	Species	3,734	1,576
SCAI Chemical (Kolárik *et al.* (2008))	SA	A	940	30,808	Chemicals	1,314	797
SCAI Disease (Gurulingappa *et al.* (2010))	SA	A	4,351	113,541	Diseases	2,241	1,003
Variome (Verspoor *et al.* (2013))	SA	FT	6,155	180,237	Diseases	5,925	475
					Genes / Proteins	4,552	529
					Species	182	8

Table SM 2: Overview of the gold standard NER corpora used to evaluate *HunFlair* and the competitor off-the-shelf tools in an cross-corpus setting. For each corpus we report the number of sentences and tokens as well as entity annotation statistics.

Corpora	Sentences	Tokens	Entity Type	Annotations	Unique
BioNLP2013-CG	5,994	157,109	Chemicals	2,405	841
(Pyysalo *et al.* (2013))			Diseases	2,604	624
			Genes / Proteins	7,908	2,057
			Species	1,801	306
CRAFT	26,589	776,028	Chemicals	6,780	1,031
(Bada *et al.* (2012))			Genes / Proteins	23,578	2,330
			Species	10,465	354
Plant-Disease (PDR)	1,780	49,392	Diseases	1,298	477
(Kim *et al.* (2019))					

2 Evaluation against off-the-shelf tools

The evaluation of *HunFlair* and its competitor biomedical NER tools is performed using the three corpora, CRAFT (Bada *et al.*, 2012), BioNLP13 Cancer Genetics (Pyysalo *et al.*, 2013) and plant-disease-relations (PDR) (Kim *et al.*, 2019). For the comparison with *SciSpacy* (Neumann *et al.*, 2019), we use the models *en_ner_craft_md*, *en_ner_jnlpba_md*, *en_ner_bc5cdr_md*, and *en_ner_bionlp13cg_md*[5]. However, when evaluating on a corpus which was used to train the specific *SciSpacy* model, we excluded the respective model and report the best score of the remaining models to retain a fair comparison. Due to this, neither *HunFlair* nor any of the competitor tools are trained on any of the corpora, hence the evaluation setting is similar to an application to completely unseen text. Table SM 2 highlights statistics of the used corpora.

We report F1 scores for all considered methods and tools. We designed our evaluation to minimize the assumptions made about the preprocessing of the input texts, especially with respect to tokenization and sentence splitting. Each model is given the complete abstract resp. full-text of the scientific article or patent as input for which it executes its own pre-processing pipeline. The predictions of each model are represented by text offsets. To calculate the evaluation scores, we use the gold standard text offsets and match them with the predicted offsets. We consider any predicted span as true positive that either exactly matches one gold standard annotation or differs only by one character either at the end or at the beginning. This accounts for the fact that the methods have differences in their processing of special characters, leading to small deviations in token off-sets.

Note, that this evaluation protocol differs substantially from the one used in Weber *et al.* (2019), where homogeneously preprocessed versions of the corpora were used for evaluation, leading to different offsets in many cases. Ad-

[5]Note, that we don't compare against the more general *SciSpacy* models (e.g. *en_core_sci_md* or *en_core_sci_lg*), since they do not provide entity types out-of-the-box.

ditionally, *HUNER* only outputs the entities as extracted from the tokenized text, losing non-ascii symbols and whitespace in the process. Thus, to align the predicted entities to the input text in the present evaluation, we try to match the predicted entity strings to the original text by using fuzzy matching. These are two important reasons for the fact that the results for Gene on the Craft corpus are much worse than those reported in Weber *et al.* (2019). This is supported by the fact that the difference between results from Weber *et al.* (2019) and those reported diminishes, when counting any overlap between predicted and annotated spans as a true positive (see Table SM 3).

We noticed that for some combinations of model and corpus *SciSpacy* predicts wrong entity boundaries in a large number of cases, leading to strikingly different results in the any-overlap evaluation and the more strict one. Nevertheless, also under this evaluation protocol, *HunFlair* performs better than all competitors except for Species on the BioNLP CG corpus.

Table SM 3: Cross corpus evaluation of off-the-shelf BioNER tools for the entity types Chemical (Ch), Disease (D), Gene (G) and Species (S) counting any overlap between the predicted and annotated span as a true positive. All scores are F1-measures and the best results are in bold. Delta shows the improvement over the more strict evaluation reported in the main text (Table 1). Misc displays the results of multiple taggers: tmChem for Chemical, GNormPus for Gene and Species, and DNorm for Disease.

	CRAFT			BioNLP CG				PDR
	Ch	G	S	Ch	D	G	S	D
Misc	44.86	67.52	82.02	74.36	60.04	71.06	**84.18**	86.95
Δ	1.98	2.59	0.87	2.21	4.40	2.09	3.65	6.32
SciSpacy	39.78	54.71	72.02	60.65	61.69	78.77	65.04	83.49
Δ	4.05	6.95	17.81	2.22	5.21	12.59	7.93	7.59
HUNER	46.84	65.27	84.66	72.00	59.74	79.58	71.43	78.49
Δ	3.85	14.50	0.21	4.63	4.42	8.36	3.59	4.85
HunFlair	**61.99**	**80.5**	**85.46**	**83.52**	**69.29**	**92.73**	80.15	**88.64**
Δ	2.16	6.99	0.42	1.70	4.22	5.02	3.74	5.20

3 Evaluation against state-of-the-art models

We compare *HunFlair* to the reported scores of the state-of-the-art models *BioBERT* (Lee *et al.*, 2019), *SciBERT* (Beltagy *et al.*, 2019), *CollaboNet* (Yoon *et al.*, 2019) and *SciSpacy* (Neumann *et al.*, 2019) on JNLPBA (only using Gene annotations), NCBI Disease and BioCreative V CDR. To obtain results that are comparable to the reported scores of these methods, we use the preprocessed versions of the corpora provided by by Lee *et al.* (2019). For this experiment, we used the large BioWordVec embeddings[6] (Chen *et al.*, 2018) and remove the

[6]`https://ftp.ncbi.nlm.nih.gov/pub/lu/Suppl/BioSentVec/BioWordVec_PubMed_MIMICIII_d200.vec.bin`

Table SM 4: Comparison with the reported results of state-of-the-art models for BioNER. Scores are macro-averaged F1 and best results are printed in bold. 'HunFlair (no)' refers to the HunFlair model without pretraining on goldstandard corpora.

	JNLPBA (Gene)	BC5CDR	NCBI
SciBERT	77.28	90.01	88.57
BioBERT v1.1	77.49	89.76	**89.71**
CollaboNET	**78.58**	87.68	88.60
SciSpacy	-	83.92	81.56
HunFlair	77.6	89.65	88.65
HunFlair (no)	77.78	**90.57**	87.47

three evaluation corpora from the pretraining set.

4 Effects of pretraining

We investigate the effects of pretraining our tagger on multiple goldstandard corpora, by comparing the pretrained tagger to a randomly initialized LSTM. Note, that the randomly initialized LSTM still uses pretrained *Flair* and *fastText* embeddings. For this experiment, we used the large BioWordVec embeddings and do not use the test portions of the corpora for pretraining. The results can be found in Table SM 5.

Pretraining improves the average results for all entity types with gains ranging from 0.8 pp for chemicals to 4.75 pp for cell lines. Performance improvements are mainly attributed to better recall. In 28 of the 34 cases the recall of the pretrained model is higher than the vanilla one. For eight cases recall improves by over 4.0 pp. This indicates that the increased amount of training data indeed leads to a better coverage of existing entities and their various surface forms as well as a higher adaptability to other biomedical subdomains. However, there are also six cases where the $F1$ score decreases slightly (max. 1.05 pp). In five out of these six cases there is a decline in precision. Additionally, also in ten cases in which $F1$ increases, precision is lower. This suggests that the larger number of entities seen in training may occasionally lead to few imprecise predictions.

Table SM 5: Comparison of the tagger that was pretrained on multiple gold standard corpora (Pretrained) vs a tagger without pretraining (Vanilla). The Δ-columns report the gains achieved through pretraining.

	Vanilla			Pretrained					
	Prec.	Rec.	F1	Prec.	Rec.	F1	ΔPrec.	ΔRec.	ΔF1
Cell Line									
CellFinder	0.9174	0.7634	0.8333	0.8983	0.8092	0.8514	-0.0191	0.0458	0.0181
CLL	0.7093	0.7922	0.7485	0.8158	0.8052	0.8105	0.1065	0.0130	0.0620
Gellus	0.7818	0.6964	0.7366	0.9375	0.7895	0.8571	0.1557	0.0931	0.1205
JLNPBA	0.7456	0.6876	0.7154	0.7485	0.6661	0.7049	0.0029	-0.0215	-0.0105
avg.	0.7885	0.7349	0.7585	0.8500	0.7675	0.8060	0.0711	0.0433	0.0528
Chemical									
BC5CDR	0.9365	0.9391	0.9378	0.9394	0.9411	0.9403	0.0029	0.0020	0.0025
CHEMDNER patent	0.8491	0.9135	0.8801	0.8471	0.9187	0.8815	-0.0020	0.0052	0.0014
CHEBI	0.8006	0.7878	0.7941	0.8220	0.7786	0.7997	0.0214	-0.0092	0.0056
CHEMDNER	0.9319	0.9171	0.9245	0.9310	0.9198	0.9254	-0.0009	0.0027	0.0009
SCAI Chemical	0.8131	0.7307	0.7697	0.8505	0.8347	0.8425	0.0374	0.1040	0.0728
avg.	0.8662	0.8576	0.8612	0.8780	0.8786	0.8779	0.0129	0.0246	0.0166
Disease									
BC5CDR	0.8615	0.8727	0.8670	0.8488	0.8804	0.8643	-0.0127	0.0077	-0.0027
miRNA	0.8318	0.8220	0.8269	0.8467	0.8769	0.8615	0.0149	0.0549	0.0346
NCBI Disease	0.8583	0.8990	0.8782	0.8663	0.8815	0.8738	0.0080	-0.0175	-0.0044
SCAI Disease	0.8159	0.7930	0.8043	0.8311	0.7972	0.8138	0.0152	0.0042	0.0095
Variome	0.9147	0.9127	0.9137	0.9072	0.9163	0.9117	-0.0075	0.0036	-0.0020
avg.	0.8564	0.8599	0.8580	0.8600	0.8705	0.8650	0.0117	0.0176	0.0106
Gene									
BioCreative II GM	0.8330	0.8284	0.8307	0.8372	0.8285	0.8328	0.0042	0.0001	0.0021
BioInfer	0.8647	0.8351	0.8497	0.8813	0.8717	0.8765	0.0166	0.0366	0.0268
CellFinder	0.8254	0.7045	0.7602	0.9050	0.8662	0.8852	0.0796	0.1617	0.1250
CHEBI	0.7811	0.6667	0.7194	0.7810	0.7155	0.7468	-0.0001	0.0488	0.0274
DECA	0.7200	0.7388	0.7293	0.7390	0.7306	0.7348	0.0190	-0.0082	0.0055
FSU-PRGE	0.9036	0.9171	0.9103	0.9020	0.9187	0.9103	-0.0016	0.0016	0.0000
CHEMDNER patent	0.6828	0.8382	0.7526	0.6875	0.8423	0.7570	0.0047	0.0041	0.0044
IEPA	0.8771	0.8771	0.8771	0.8754	0.8870	0.8812	-0.0017	0.0099	0.0041
JNLPBA	0.8366	0.8561	0.8462	0.8287	0.8507	0.8396	-0.0079	-0.0054	-0.0066
LocText	0.8646	0.8202	0.8418	0.8689	0.8881	0.8784	0.0043	0.0679	0.0366
miRNA	0.7644	0.7956	0.7797	0.7541	0.8679	0.8070	-0.0103	0.0723	0.0273
OSIRIS	0.8721	0.8926	0.8823	0.9123	0.9430	0.9274	0.0402	0.0504	0.0451
Variome	0.9223	0.9482	0.9351	0.9169	0.9519	0.9340	-0.0054	0.0037	-0.0011
avg.	0.8267	0.8245	0.8242	0.8376	0.8586	0.8470	0.0150	0.0362	0.0240
Species									
CellFinder	0.8489	0.9219	0.8839	0.8414	0.9531	0.8938	-0.0075	0.0312	0.0099
CHEBI	0.8875	0.7890	0.8353	0.8807	0.7765	0.8253	-0.0068	-0.0125	-0.0100
Linneaus	0.9440	0.9142	0.9289	0.9579	0.9470	0.9524	0.0139	0.0328	0.0235
LocText	0.9545	0.9130	0.9333	0.9468	0.9674	0.9570	-0.0077	0.0544	0.0237
miRNA	0.9914	0.9312	0.9603	0.9789	0.9393	0.9587	-0.0125	0.0081	-0.0016
S800	0.7664	0.7232	0.7442	0.7396	0.7518	0.7457	-0.0268	0.0286	0.0015
Variome	0.5400	0.8182	0.6506	0.6829	0.8485	0.7568	0.1429	0.0303	0.1062
avg.	0.8475	0.8587	0.8481	0.8612	0.8834	0.8700	0.0312	0.0283	0.0252

References

Akhondi, S. A., Klenner, A. G., Tyrchan, C., Manchala, A. K., Boppana, K., Lowe, D., Zimmermann, M., Jagarlapudi, S. A., Sayle, R., Kors, J. A., *et al.* (2014). Annotated chemical patent corpus: a gold standard for text mining. *PloS one*, **9**(9), e107477.

Bada, M., Eckert, M., Evans, D., Garcia, K., Shipley, K., Sitnikov, D., Baumgartner, W. A., Cohen, K. B., Verspoor, K., Blake, J. A., *et al.* (2012). Concept annotation in the craft corpus. *BMC Bioinformatics*, **13**(1), 161.

Bagewadi, S., Bobić, T., Hofmann-Apitius, M., Fluck, J., and Klinger, R. (2014). Detecting miRNA mentions and relations in biomedical literature. *F1000Res.*, **3**.

Beltagy, I., Lo, K., and Cohan, A. (2019). SciBERT: A pretrained language model for scientific text. In *Proceedings of the 2019 Conference on Empirical Methods in Natural Language Processing and the 9th International Joint Conference on Natural Language Processing (EMNLP-IJCNLP)*, pages 3613–3618, Hong Kong, China. Association for Computational Linguistics.

Bojanowski, P., Grave, E., Joulin, A., and Mikolov, T. (2017). Enriching word vectors with subword information. *Transactions of the Association for Computational Linguistics*, **5**, 135–146.

Chen, Q., Peng, Y., and Lu, Z. (2018). Biosentvec: creating sentence embeddings for biomedical texts. *CoRR*, **abs/1810.09302**.

Ding, J., Berleant, D., Nettleton, D., and Wurtele, E. (2002). Mining MEDLINE: abstracts, sentences, or phrases? *Pac. Symp. Biocomput.*, pages 326–337.

Doğan, R. I., Leaman, R., and Lu, Z. (2014). NCBI disease corpus: a resource for disease name recognition and concept normalization. *J. Biomed. Inform.*, **47**, 1–10.

Furlong, L. I., Dach, H., Hofmann-Apitius, M., and Sanz, F. (2008). OSIRISv1.2: a named entity recognition system for sequence variants of genes in biomedical literature. *BMC Bioinformatics*, **9**, 84.

Gerner, M., Nenadic, G., and Bergman, C. M. (2010). LINNAEUS: a species name identification system for biomedical literature. *BMC Bioinformatics*, **11**, 85.

Goldberg, T., Vinchurkar, S., Cejuela, J. M., Jensen, L. J., and Rost, B. (2015). Linked annotations: a middle ground for manual curation of biomedical databases and text corpora. *BMC Proc.*, **9**(5), A4.

Gurulingappa, H., Klinger, R., Hofmann-Apitius, M., and Fluck, J. (2010). An empirical evaluation of resources for the identification of diseases and adverse effects in biomedical literature. In *2nd Workshop on Building and evaluating resources for biomedical text mining (7th edition of the Language Resources and Evaluation Conference)*. pub.uni-bielefeld.de.

Hahn, U., Tomanek, K., Beisswanger, E., and Faessler, E. (2010). A proposal for a configurable silver standard. In *Proceedings of the Fourth Linguistic Annotation Workshop*, pages 235–242. aclweb.org.

Kaewphan, S., Van Landeghem, S., Ohta, T., Van de Peer, Y., Ginter, F., and Pyysalo, S. (2016). Cell line name recognition in support of the identification of synthetic lethality in cancer from text. *Bioinformatics*, **32**(2), 276–282.

Kim, B., Choi, W., and Lee, H. (2019). A corpus of plant–disease relations in the biomedical domain. *PLOS ONE*, **14**(8), 1–19.

Kim, J.-D., Ohta, T., Tsuruoka, Y., Tateisi, Y., and Collier, N. (2004). Introduction to the bio-entity recognition task at JNLPBA. In *Proceedings of the international joint workshop on natural language processing in biomedicine and its applications*, pages 70–75. Citeseer.

Kolárik, C., Klinger, R., Friedrich, C. M., Hofmann-Apitius, M., and Fluck, J. (2008). Chemical names: terminological resources and corpora annotation. In *Workshop on Building and evaluating resources for biomedical text mining (6th edition of the Language Resources and Evaluation Conference)*. pub.uni-bielefeld.de.

Krallinger, M., Rabal, O., Leitner, F., Vazquez, M., Salgado, D., Lu, Z., Leaman, R., Lu, Y., Ji, D., Lowe, D. M., Sayle, R. A., Batista-Navarro, R. T., Rak, R., Huber, T., Rocktäschel, T., Matos, S., Campos, D., Tang, B., Xu, H., Munkhdalai, T., Ryu, K. H., Ramanan, S. V., Nathan, S., Žitnik, S., Bajec, M., Weber, L., Irmer, M., Akhondi, S. A., Kors, J. A., Xu, S., An, X., Sikdar, U. K., Ekbal, A., Yoshioka, M., Dieb, T. M., Choi, M., Verspoor, K., Khabsa, M., Giles, C. L., Liu, H., Ravikumar, K. E., Lamurias, A., Couto, F. M., Dai, H.-J., Tsai, R. T.-H., Ata, C., Can, T., Usié, A., Alves, R., Segura-Bedmar, I., Martínez, P., Oyarzabal, J., and Valencia, A. (2015a). The CHEMDNER corpus of chemicals and drugs and its annotation principles. *J. Cheminform.*, **7**(Suppl 1 Text mining for chemistry and the CHEMDNER track), S2.

Krallinger, M., Rabal, O., Lourenço, A., Perez, M. P., Rodriguez, G. P., Vazquez, M., Leitner, F., Oyarzabal, J., and Valencia, A. (2015b). Overview of the CHEMDNER patents task. In *Proceedings of the fifth BioCreative challenge evaluation workshop*, pages 63–75. biocreative.bioinformatics.udel.edu.

Lee, J., Yoon, W., Kim, S., Kim, D., Kim, S., So, C. H., and Kang, J. (2019). BioBERT: a pre-trained biomedical language representation model for biomedical text mining. *Bioinformatics*. btz682.

Li, J., Sun, Y., Johnson, R. J., Sciaky, D., Wei, C.-H., Leaman, R., Davis, A. P., Mattingly, C. J., Wiegers, T. C., and Lu, Z. (2016). BioCreative V CDR task corpus: a resource for chemical disease relation extraction. *Database*, **2016**.

Neumann, M., King, D., Beltagy, I., and Ammar, W. (2019). ScispaCy: Fast and robust models for biomedical natural language processing. In *Proceedings of the 18th BioNLP Workshop and Shared Task*, pages 319–327, Florence, Italy. Association for Computational Linguistics.

Neves, M., Damaschun, A., Kurtz, A., and Leser, U. (2012). Annotating and evaluating text for stem cell research. In *Proceedings of the Third Workshop on Building and Evaluation Resources for Biomedical Text Mining (BioTxtM 2012) at Language Resources and Evaluation (LREC). Istanbul, Turkey*, pages 16–23. Citeseer.

Pafilis, E., Frankild, S. P., Fanini, L., Faulwetter, S., Pavloudi, C., Vasileiadou, A., Arvanitidis, C., and Jensen, L. J. (2013). The SPECIES and ORGANISMS resources for fast and accurate identification of taxonomic names in text. *PLoS One*, **8**(6), e65390.

Pérez-Pérez, M., Rabal, O., Pérez-Rodríguez, G., Vazquez, M., Fdez-Riverola, F., Oyarzabal, J., Valencia, A., Lourenço, A., and Krallinger, M. (2017). Evaluation of chemical and gene/protein entity recognition systems at biocreative v. 5: the cemp and gpro patents tracks.

Pyysalo, S., Ginter, F., Heimonen, J., Björne, J., Boberg, J., Järvinen, J., and Salakoski, T. (2007). BioInfer: a corpus for information extraction in the biomedical domain. *BMC Bioinformatics*, **8**, 50.

Pyysalo, S., Ohta, T., and Ananiadou, S. (2013). Overview of the cancer genetics (CG) task of BioNLP shared task 2013. In *Proceedings of the BioNLP Shared Task 2013 Workshop*, pages 58–66, Sofia, Bulgaria. Association for Computational Linguistics.

Shardlow, M. J., Nguyen, N., Owen, G., O'Donovan, C., Leach, A., McNaught, J., Turner, S., and Ananiadou, S. (2018). A new corpus to support text mining for the curation of metabolites in the ChEBI database. In *Proceedings of the Eleventh International Conference on Language Resources and Evaluation (LREC 2018)*, pages 280–285. e-space.mmu.ac.uk.

Smith, L., Tanabe, L. K., Ando, R. J. N., Kuo, C.-J., Chung, I.-F., Hsu, C.-N., Lin, Y.-S., Klinger, R., Friedrich, C. M., Ganchev, K., Torii, M., Liu, H., Haddow, B., Struble, C. A., Povinelli, R. J., Vlachos, A., Baumgartner, Jr, W. A., Hunter, L., Carpenter, B., Tsai, R. T.-H., Dai, H.-J., Liu, F., Chen, Y., Sun, C., Katrenko, S., Adriaans, P., Blaschke, C., Torres, R., Neves, M., Nakov, P., Divoli, A., Maña-López, M., Mata, J., and Wilbur, W. J. (2008). Overview of BioCreative II gene mention recognition. *Genome Biol.*, **9 Suppl 2**, S2.

Verspoor, K., Jimeno Yepes, A., Cavedon, L., McIntosh, T., Herten-Crabb, A., Thomas, Z., and Plazzer, J.-P. (2013). Annotating the biomedical literature for the human variome. *Database*, **2013**, bat019.

Wang, X., Tsujii, J., and Ananiadou, S. (2010). Disambiguating the species of biomedical named entities using natural language parsers. *Bioinformatics*, **26**(5), 661–667.

Weber, L., Münchmeyer, J., Rocktäschel, T., Habibi, M., and Leser, U. (2019). HUNER: improving biomedical NER with pretraining. *Bioinformatics*, **36**(1), 295–302.

Yoon, W., So, C. H., Lee, J., and Kang, J. (2019). Collabonet: collaboration of deep neural networks for biomedical named entity recognition. *BMC Bioinformatics*, **20-S**(10), 55–65.
